# Supplementary material for: Behavioural risk factors for cardiovascular diseases among adolescents of secondary school in Tulsipur Sub-Metropolitan City, Nepal: A cross-sectional study
Source: PLoS One. 2025 Sep 11;20(9):e0313943. doi: 10.1371/journal.pone.0313943 (PMC12425188; doi:10.1371/journal.pone.0313943)
Supplement: S1 Appendix — (DOCX) [file pone.0313943.s001.docx]

## **Current smoker/ smokeless tobacco user/ alcohol user**: Those who are using the products in past 30 days preceding the study [1].

Inadequate fruit and/ or vegetable intake: Less than five servings of fruits and/or vegetables a day was considered inadequate [1].

Added salt intake: It was defined for study participants who identified to add dietary salt to food always or often during eating [1].

Consumption of processed food high in salt: Processed food high in salt means foods that have been altered from their natural state, such as packaged salty locally available snacks, canned salty food, salty food prepared at fast food restaurants, cheese, processed meat, etc. Consumption of processed food high in salt was defined for those participants who used to consume processed food high in salt always or often [1].

Adequate physical activity: Adequate physical activity was defined as moderate to vigorous activity greater than or equal to 60 min/day for all 7 days of previous week [1].

Sedentary behaviour: Sedentary behaviour included spending time in school, watching television and mobiles and playing video games. Spending time only by sitting or lying down for 9 hours or more, per day as defined as sedentary behaviour [2].

**REFERENCES:**

1. Dhimal M, Bista B, Bhattarai S, Dixit LP, Hyder MKA, Agrawal N, et al. Report of non-communicable disease risk factors: STEPS survey Nepal 2019. Kathmandu: Nepal Health Research Council; 2020.
2. Islam TMM, Banik PC, Barua L, Islam SMS, Chowdhury S, Ahmed MSAM. Cardiovascular disease risk factors among school children of Bangladesh: a cross-sectional study. BMJ Open . 2020 Oct;10(10):e038077.
