# Supplementary material for: Behavioural risk factors for cardiovascular diseases among adolescents of secondary school in Tulsipur Sub-Metropolitan City, Nepal: A cross-sectional study
Source: PLoS One. 2025 Sep 11;20(9):e0313943. doi: 10.1371/journal.pone.0313943 (PMC12425188; doi:10.1371/journal.pone.0313943)
Supplement: S1 Table — (DOCX) [file pone.0313943.s003.docx]

The model fitness test was assessed using overall accuracy, Nagelkerke R² and the Hosmer-Lemeshow goodness-of-fit test as presented in Supplementary table 1. Model 1 exhibited improved classification accuracy compared to the null model across all risk factors with highest accuracy observed for **caloric drink intake (99.1%),** followed by **current smokeless tobacco users (91.3%)** and **current alcohol users (89.9%).** The Nagelkerke R² indicated highest predictive power for **caloric drink intake (Nagelkerke R² = 0.738),** suggesting strong explanatory capability. The Hosmer-Lemeshow test indicated a good model fit with all models meeting criterion, suggesting that the predicted probabilities closely matched the observed data.

**Table 1 Summary of binary logistics regression analysis across different dependent variables**

| **Category** | **Overall Accuracy** | | **Nagelkerke R²** | **Hosmer-Lemeshow Test (p-value)** |
| --- | --- | --- | --- | --- |
|  | **Null Model** | **Model 1** |  |  |
| **Processed Food** | 66.1 | 70.7 | .121 | >0.05 |
| **Caloric Drink** | 98.8 | 99.1 | .738 | >0.05 |
| **Added Salt** | 84.1 | 84.3 | .054 | >0.05 |
| **Sedentary Behavior** | 60.0 | 64,6 | 0.121 | >0.05 |
| **Insufficient Fruit and Vegetable Intake** | 57.7 | 62.0 | 0.071 | >0.05 |
| **Physical inactivity** | 64.6 | 65.2 | 0.103 | >0.05 |
| **Refined Vegetable Oil Use** | 80.9 | 82.0 | 0.160 | >0.05 |
| **Current Smoker** | 87.5 | 89.0 | 0.345 | >0.05 |
| **Current Alcohol users** | 89.6 | 89.9 | 0.280 | >0.05 |
| **Current Smokeless tobacco users** | 91.3 | 91.3 | 0.199 | >0.05 |

**Table 2: Association and odds of processed food consumption with sociodemographic characteristics**

| Characteristics | Categories | Processed food consumption (always or often) n (%) | B (SE) | p-value | OR  Exp(B) | 95% CI |
| --- | --- | --- | --- | --- | --- | --- |
| Age | 16-17 | 63(36.4%) | Reference | | | |
|  | 18-19 | 56(29.8%) | -.046 (.259) | .860 | .955 | [.575, 1.586] |
| Gender^+^ | Male | 47(28.7%) | -0.516 (0.256) | 0.044 | 0.597 | [0.362, 0.986] |
|  | Female | 72(36.9%) | Reference | | | |
| Ethnicity | Brahmin/Chhetri | 93(36.5%) | 0.775 (0.288) | 0.007 | 2.171 | [1.234, 3.821] |
|  | Others | 26(24.5%) | Reference | | | |
| Religion | Hindu | 116(32.8%) | -1.192 (0.846) | 0.159 | 0.304 | [0.058, 1.592] |
|  | Others | 3(42.9%) | Reference | | | |
| Grade | 11 | 72(39.6%) | Reference | | | |
|  | 12 | 47(26.3%) | -0.602 (0.255) | 0.018 | 0.548 | [0.332, 0.903] |
| Education System | Public | 43(35.8%) | Reference | | | |
|  | Private | 76(31.5%) | -0.537 (0.277) | 0.052 | 0.584 | [0.340, 1.005] |
| Current living condition | Living with family | 87 (31.8%) | -0.197 (0.285) | 0.489 | 0.821 | [0.469, 1.436] |
|  | Others | 32(36.8%) | Reference | | | |
| Mother’s* education | No schooling | 20(22.2%) | Reference | | | |
|  | Formal schooling | 98(36.7%) | 0.1063 (0.354) | 0.003 | 2.895 | [1.447, 5.793] |
| Mother’s* occupation | Employed | 116(33.0%) | -0.106 (0.253) | 0.675 | 0.899 | [0.548, 1.477] |
|  | Unemployed | 2(40.0%) | Reference | | | |
| Father’s** education | No schooling | 13(34.2%) | Reference | | | |
|  | Formal schooling | 105(33.7%) | -0.392 (0.442) | 0.375 | 0.676 | [0.284, 1.606] |
| Father’s** occupation | Employed | 114(34.1%) | 0.451 (0.621) | 0.468 | 1.570 | [0.465, 5.308] |
|  | Unemployed | 4(25.0%) | Reference | | | |
| Parents’*** chronic illness | Yes | 22(31.4%) | 0.034 (0.312) | 0.913 | 1.035 | [0.561, 1.909] |
|  | No | 96(33.3%) | Reference | | | |
| Parent’s*** smoking habit | Yes | 15(28.3%) | -0.437 (0.414) | 0.291 | 0.646 | [0.287, 1.453] |
|  | No | 102(33.4%) | Reference | | | |
| Parent’s*** smokeless tobacco | Yes | 31(35.2%) | 0.249 (0.315) | 0.430 | 1.282 | [0.691, 2.377] |
|  | No | 86(31.9%) | Reference | | | |
| Parent’s*** drinking habit | Yes | 34(34.7%) | 0.463 (0.316) | 0.143 | 1.589 | [0.856, 2.949] |
|  | No | 83(31.9%) | Reference | | | |

**Table 2: Association and odds of caloric drink consumption with sociodemographic characteristics**

| Characteristics | Categories | Calorie Drink Consumption (Yes) n (%) | B (SE) | p-value | OR  Exp(B) | 95% CI |  |  |  |
| --- | --- | --- | --- | --- | --- | --- | --- | --- | --- |
| Age | 16-17 | 170 (98.3%) | Reference | | | |  |  |  |
|  | 18-19 | 186 (98.9%) | -38.567 (1151.450) | 0.973 | 0.00 | [0.00, .] |  |  |  |
| Gender^+^ | Male | 164 (100%) | 53.899 (2248.667) | 0.981 | 2.56E+23 | [0.00, .] |  |  |  |
|  | Female | 190 (97.4%) | Reference | | | |  |  |  |
| Ethnicity | Brahmin/Chhetri | 250 (98.0%) | -54.122 (2308.374) | 0.981 | 0.00 | [0.00, .] |  |  |  |
|  | Others | 106 (100%) | Reference | | | |  |  |  |
| Religion | Hindu | 349 (98.6%) | 35.230 (11756.759) | 0.998 | 1.99E+15 | [0.00, .] |  |  |  |
|  | Others | 7 (100%) | Reference | | | |  |  |  |
| Grade | 11 | 32 (100%) | Reference | | | |  |  |  |
|  | 12 | 68 (100%) | 66.224 (2378.508) | 0.978 | 5.76E+28 | [0.00, .] |  |  |  |
| Education System | Public | 119 (99.2%) | Reference | | | |  |  |  |
|  | Private | 237 (98.3%) | 13.121 (463.255) | 0.977 | 499108.22 | [0.00, .] |  |  |  |
| Current living condition | Living with family | 271 (98.9%) | 25.807 (817.684) | 0.975 | 1.61E+11 | [0.00, .] |  |  |  |
|  | Others | 85 (97.7%) | Reference | | | |  |  |  |
| Mother’s* education | No formal schooling | 89 (98.9%) | Reference | | | |  |  |  |
|  | Formal schooling | 264 (98.9%) | -12.413 (463.256) | 0.979 | 0.00 | [0.00, .] |  |  |  |
| Mother’s* occupation | Employed | 348 (98.9%) | 79.839 (2680.583) | 0.976 | 4.72E+34 | [0.00, .] |  |  |  |
|  | Unemployed | 5 (100%) | Reference | | | |  |  |  |
| Father’s** education | No formal schooling | 38 (100%) | Reference | | | |  |  |  |
|  | Formal schooling | 308 (98.7%) | -55.602 (4206.810) | 0.989 | 0.00 | [0.00, .] |  |  |  |
| Father’s** occupation | Employed | 330 (98.8%) | 10.922 (6519.604) | 0.999 | 55399.72 | [0.00, .] |  |  |  |
|  | Unemployed | 16 (100%) | Reference | | | |  |  |  |
| Parents’*** chronic illness | Yes | 69 (98.6%) | -12.773 (463.254) | 0.978 | 0.00 | [0.00, .] |  |  |  |
|  | No | 285 (99.0%) | Reference | | | |  |  |  |
| Parent’s*** smoking habit | Yes | 51 (96.2%) | -39.275 (1151.450) | 0.973 | 0.00 | [0.00, .] |  |  |  |
|  | No | 303 (99.3%) | Reference | | | |  |  |  |
| Parent’s*** smokeless tobacco | Yes | 86 (97.7%) | 0.579 (1.887) | 0.759 | 1.78 | [0.04, 72.13] |  |  |  |
|  | No | 268 (99.3%) | Reference | | | |  |  |  |
| Parent’s*** drinking habit | Yes | 96 (98.0%) | -0.116 (2.195) | 0.958 | 0.89 | [0.01, 65.83] |  |  |  |
|  | No | 258 (99.2%) | Reference | | | |  |  |  |

**Table 3: Association and odds of added salt intake with sociodemographic characteristics**

| Characteristics | Categories | Added Salt Intake (Always or Often) n (%) | B (SE) | p-value | OR  Exp(B) | 95% CI |  |  |  |
| --- | --- | --- | --- | --- | --- | --- | --- | --- | --- |
| Age | 16-17 | 26 (15.0%) | Reference | | | |  |  |  |
|  | 18-19 | 30 (16.0%) | 0.101 (0.326) | 0.757 | 1.106 | [0.584, 2.095] |  |  |  |
| Gender^+^ | Male | 25 (15.2%) | -0.022 (0.318) | 0.946 | 0.979 | [0.525, 1.825] |  |  |  |
|  | Female | 31 (15.9%) | Reference | | | |  |  |  |
| Ethnicity | Brahmin/Chhetri | 40 (15.7%) | 0.226 (0.348) | 0.517 | 1.253 | [0.634, 2.477] |  |  |  |
|  | Others | 16 (15.1%) | Reference | | | |  |  |  |
| Religion | Hindu | 54 (15.3%) | -0.623 (0.899) | 0.489 | 0.536 | [0.092, 3.127] |  |  |  |
|  | Others | 2 (28.6%) | Reference | | | |  |  |  |
| Grade | 11 | 33 (18.1%) | Reference | | | |  |  |  |
|  | 12 | 23 (12.8%) | -0.436 (0.324) | 0.178 | 0.646 | [0.343, 1.219] |  |  |  |
| Education System | Public | 21 (17.5%) | Reference | | | |  |  |  |
|  | Private | 35 (14.5%) | -0.251 (0.339) | 0.458 | 0.778 | [0.400, 1.511] |  |  |  |
| Current living condition | Living with family | 43 (15.7%) | 0.274 (0.374) | 0.464 | 1.315 | [0.632, 2.735] |  |  |  |
|  | Others | 13 (14.9%) | Reference | | | |  |  |  |
| Mother’s* education | No formal schooling | 20 (22.2%) | Reference | | | |  |  |  |
|  | Formal schooling | 36 (13.5%) | -0.585 (0.366) | 0.110 | 0.557 | [0.272, 1.141] |  |  |  |
| Mother’s* occupation | Employed | 54 (15.3%) | 0.229 (0.315) | 0.467 | 1.257 | [0.678, 2.332] |  |  |  |
|  | Unemployed | 2 (40.0%) | Reference | | | |  |  |  |
| Father’s** education | No formal schooling | 7 (18.4%) | Reference | | | |  |  |  |
|  | Formal schooling | 48 (15.4%) | 0.275 (0.510) | 0.590 | 1.316 | [0.484, 3.574] |  |  |  |
| Father’s** occupation | Employed | 51 (15.3%) | -0.648 (0.631) | 0.305 | 0.523 | [0.152, 1.802] |  |  |  |
|  | Unemployed | 4 (25.0%) | Reference | | | |  |  |  |
| Parents’*** chronic illness | Yes | 8 (11.4%) | -0.495 (0.426) | 0.245 | 0.610 | [0.265, 1.404] |  |  |  |
|  | No | 48 (16.7%) | Reference | | | |  |  |  |
| Parent’s*** smoking habit | Yes | 11 (20.8%) | 0.362 (0.471) | 0.442 | 1.437 | [0.570, 3.619] |  |  |  |
|  | No | 45 (14.8%) | Reference | | | |  |  |  |
| Parent’s*** smokeless tobacco | Yes | 16 (18.2%) | 0.109 (0.396) | 0.784 | 1.115 | [0.513, 2.424] |  |  |  |
|  | No | 40 (14.8%) | Reference | | | |  |  |  |
| Parent’s*** drinking habit | Yes | 16 (16.3%) | -0.044 (0.399) | 0.913 | 0.957 | [0.438, 2.094] |  |  |  |
|  | No | 40 (15.4%) | Reference | | | |  |  |  |

Table 4

| Characteristics | Categories | Sedentary Behaviour | B (SE) | p-value | OR  Exp(B) | 95% CI |  |  |  |
| --- | --- | --- | --- | --- | --- | --- | --- | --- | --- |
| Age | 16-17 | 34 (56.7%) | Reference | | | |  |  |  |
|  | 18-19 | 43 (38.1%) | 0.110 (0.252) | 0.661 | 1.117 | [0.682, 1.829] |  |  |  |
| Gender^+^ | Male | 98 (59.8%) | -0.128 (0.244) | 0.599 | 0.880 | [0.546, 1.419] |  |  |  |
|  | Female | 117 (60.0%) | Reference | | | |  |  |  |
| Ethnicity | Brahmin/Chhetri | 156 (61.2%) | 0.350 (0.265) | 0.185 | 1.420 | [0.845, 2.384] |  |  |  |
|  | Others | 61 (57.5%) | Reference | | | |  |  |  |
| Religion | Hindu | 212 (59.9%) | -0.976 (0.913) | 0.285 | 0.377 | [0.063, 2.258] |  |  |  |
|  | Others | 5 (71.4%) | Reference | | | |  |  |  |
| Grade | 11 | 104 (57.1%) | Reference | | | |  |  |  |
|  | 12 | 113 (63.1%) | 0.261 (0.245) | 0.286 | 1.298 | [0.803, 2.097] |  |  |  |
| Education System | Public | 85 (70.8%) | Reference | | | |  |  |  |
|  | Private | 132 (54.8%) | -1.048 (0.282) | 0.000 | 0.351 | [0.202, 0.609] |  |  |  |
| Current living condition | Living with family | 163 (59.5%) | -0.020 (0.279) | 0.944 | 0.980 | [0.568, 1.694] |  |  |  |
|  | Others | 54 (62.1%) | Reference | | | |  |  |  |
| Mother’s* education | No formal schooling | 51 (56.7%) | Reference | | | |  |  |  |
|  | Formal schooling | 164 (61.4%) | 0.189 (0.305) | 0.536 | 1.208 | [0.664, 2.197] |  |  |  |
| Mother’s* occupation | Employed | 78 (63.4%) | 0.031 (0.246) | 0.899 | 1.032 | [0.637, 1.670] |  |  |  |
|  | Unemployed | 139 (58.4%) | Reference | | | |  |  |  |
| Father’s** education | No formal schooling | 18 (47.4%) | Reference | | | |  |  |  |
|  | Formal schooling | 194 (62.2%) | 0.569 (0.403) | 0.157 | 1.767 | [0.803, 3.889] |  |  |  |
| Father’s** occupation | Employed | 203 (60.8%) | 0.216 (0.549) | 0.694 | 1.241 | [0.423, 3.636] |  |  |  |
|  | Unemployed | 9 (56.3%) | Reference | | | |  |  |  |
| Parents’*** chronic illness | Yes | 43 (61.4%) | 0.057 (0.301) | 0.849 | 1.059 | [0.587, 1.908] |  |  |  |
|  | No | 172 (59.7%) | Reference | | | |  |  |  |
| Parent’s*** smoking habit | Yes | 28 (52.8%) | -0.372 (0.386) | 0.336 | 0.690 | [0.324, 1.469] |  |  |  |
|  | No | 188 (61.6%) | Reference | | | |  |  |  |
| Parent’s*** smokeless tobacco | Yes | 46 (47.7%) | -0.673 (0.306) | 0.028 | 0.510 | [0.280, 0.930] |  |  |  |
|  | No | 100 (37.0%) | Reference | | | |  |  |  |
| Parent’s*** drinking habit | Yes | 64 (65.3%) | 0.867 (0.318) | 0.006 | 2.380 | [1.275, 4.442] |  |  |  |
|  | No | 152 (58.5%) | Reference | | | |  |  |  |

Table 5

| Characteristics | Categories | Insufficient Fruit and Vegetable Intake | B (SE) | p-value | OR  Exp(B) | 95% CI |  |  |  |
| --- | --- | --- | --- | --- | --- | --- | --- | --- | --- |
| Age | 16-17 | 96 (55.5%) | Reference | | | |  |  |  |
|  | 18-19 | 110 (58.5%) | 0.089 (0.246) | 0.717 | 1.093 | [0.675, 1.771] |  |  |  |
| Gender^+^ | Male | 95 (57.9%) | 0.036 (0.238) | 0.881 | 1.036 | [0.650, 1.652] |  |  |  |
|  | Female | 110 (56.4%) | Reference | | | |  |  |  |
| Ethnicity | Brahmin/Chhetri | 144 (56.5%) | -0.045 (0.259) | 0.861 | 0.956 | [0.576, 1.587] |  |  |  |
|  | Others | 62 (58.5%) | Reference | | | |  |  |  |
| Religion | Hindu | 203 (57.3%) | 0.410 (0.807) | 0.612 | 1.506 | [0.309, 7.333] |  |  |  |
|  | Others | 3 (42.9%) | Reference | | | |  |  |  |
| Grade | 11 | 98 (53.8%) | Reference | | | |  |  |  |
|  | 12 | 108 (60.3%) | 0.221 (0.239) | 0.355 | 1.247 | [0.781, 1.991] |  |  |  |
| Education System | Public | 76 (63.3%) | Reference | | | |  |  |  |
|  | Private | 130 (53.9%) | -0.656 (0.265) | 0.013 | 0.519 | [0.309, 0.873] |  |  |  |
| Current living condition | Living with family | 155 (56.6%) | -0.013 (0.273) | 0.961 | 0.987 | [0.578, 1.686] |  |  |  |
|  | Others | 51 (58.6%) | Reference | | | |  |  |  |
| Mother’s* education | No formal schooling | 44 (48.9%) | Reference | | | |  |  |  |
|  | Formal schooling | 159 (59.6%) | 0.355 (0.295) | 0.229 | 1.426 | [0.800, 2.544] |  |  |  |
| Mother’s* occupation | Employed | 75 (61.0%) | 0.205 (0.239) | 0.393 | 1.227 | [0.767, 1.962] |  |  |  |
|  | Unemployed | 131 (55.0%) | Reference | | | |  |  |  |
| Father’s** education | No formal schooling | 18 (47.4%) | Reference | | | |  |  |  |
|  | Formal schooling | 184 (59.0%) | 0.307 (0.394) | 0.436 | 1.359 | [0.628, 2.942] |  |  |  |
| Father’s** occupation | Employed | 196 (58.7%) | 0.895 (0.546) | 0.101 | 2.447 | [0.840, 7.129] |  |  |  |
|  | Unemployed | 6 (37.5%) | Reference | | | |  |  |  |
| Parents’*** chronic illness | Yes | 37 (52.9%) | -0.203 (0.288) | 0.481 | 0.817 | [0.465, 1.435] |  |  |  |
|  | No | 168 (58.3%) | Reference | | | |  |  |  |
| Parent’s*** smoking habit | Yes | 26 (49.1%) | -0.542 (0.374) | 0.147 | 0.582 | [0.279, 1.211] |  |  |  |
|  | No | 179 (58.7%) | Reference | | | |  |  |  |
| Parent’s*** smokeless tobacco | Yes | 54 (61.4%) | 0.465 (0.307) | 0.130 | 1.591 | [0.873, 2.902] |  |  |  |
|  | No | 151 (55.9%) | Reference | | | |  |  |  |
| Parent’s*** drinking habit | Yes | 55 (56.1%) | -0.007 (0.296) | 0.982 | 0.993 | [0.557, 1.773] |  |  |  |
|  | No | 150 (57.7%) | Reference | | | |  |  |  |

Table 6

| Characteristics | Categories | Physical Inactivity | B (SE) | p-value | OR  Exp(B) | 95% CI |  |  |  |
| --- | --- | --- | --- | --- | --- | --- | --- | --- | --- |
| Age | 16-17 | 54 (31.2%) | Reference | | | |  |  |  |
|  | 18-19 | 72 (38.3%) | 0.200 (0.259) | 0.439 | 1.222 | [0.736, 2.029] |  |  |  |
| Gender^+^ | Male | 62 (37.8%) | 0.114 (0.248) | 0.646 | 1.121 | [0.690, 1.822] |  |  |  |
|  | Female | 62 (31.8%) | Reference | | | |  |  |  |
| Ethnicity | Brahmin/Chhetri | 92 (36.1%) | -0.036 (0.274) | 0.896 | 0.965 | [0.564, 1.651] |  |  |  |
|  | Others | 34 (32.1%) | Reference | | | |  |  |  |
| Religion | Hindu | 123 (34.7%) | -0.187 (0.819) | 0.819 | 0.829 | [0.167, 4.126] |  |  |  |
|  | Others | 3 (42.9%) | Reference | | | |  |  |  |
| Grade | 11 | 58 (31.9%) | Reference | | | |  |  |  |
|  | 12 | 68 (38.0%) | 0.045 (0.249) | 0.856 | 1.046 | [0.642, 1.704] |  |  |  |
| Education System | Public | 24 (20.0%) | Reference | | | |  |  |  |
|  | Private | 102 (42.3%) | 1.033 (0.295) | 0.000 | 2.810 | [1.574, 5.013] |  |  |  |
| Current living condition | Living with family | 98 (35.8%) | -0.184 (0.286) | 0.519 | 0.832 | [0.475, 1.456] |  |  |  |
|  | Others | 28 (32.2%) | Reference | | | |  |  |  |
| Mother’s* education | No formal schooling | 46 (25.6%) | Reference | | | |  |  |  |
|  | Formal schooling | 103 (38.6%) | 0.247 (0.320) | 0.441 | 1.280 | [0.683, 2.397] |  |  |  |
| Mother’s* occupation | Employed | 46 (37.4%) | 0.059 (0.247) | 0.812 | 1.061 | [0.653, 1.723] |  |  |  |
|  | Unemployed | 80 (33.6%) | Reference | | | |  |  |  |
| Father’s** education | No formal schooling | 8 (21.1%) | Reference | | | |  |  |  |
|  | Formal schooling | 117 (37.5%) | 0.467 (0.461) | 0.311 | 1.596 | [0.647, 3.938] |  |  |  |
| Father’s** occupation | Employed | 118 (35.3%) | -0.525 (0.561) | 0.350 | 0.592 | [0.197, 1.777] |  |  |  |
|  | Unemployed | 7 (43.8%) | Reference | | | |  |  |  |
| Parents’*** chronic illness | Yes | 24 (34.3%) | -0.201 (0.307) | 0.513 | 0.818 | [0.448, 1.494] |  |  |  |
|  | No | 102 (35.4%) | Reference | | | |  |  |  |
| Parent’s*** smoking habit | Yes | 18 (34.0%) | -0.335 (0.396) | 0.398 | 0.715 | [0.329, 1.554] |  |  |  |
|  | No | 107 (35.1%) | Reference | | | |  |  |  |
| Parent’s*** smokeless tobacco | Yes | 34 (38.6%) | 0.195 (0.310) | 0.530 | 1.215 | [0.661, 2.232] |  |  |  |
|  | No | 91 (33.7%) | Reference | | | |  |  |  |
| Parent’s*** drinking habit | Yes | 41 (41.8%) | 0.371 (0.302) | 0.219 | 1.449 | [0.802, 2.619] |  |  |  |
|  | No | 84 (32.3%) | Reference | | | |  |  |  |

Table 7

| Characteristics | Categories | Refined Vegetable Oil User n (%) | B (SE) | p-value | OR  Exp(B) | 95% CI |  |  |  |
| --- | --- | --- | --- | --- | --- | --- | --- | --- | --- |
| Age | 16-17 | 34 (19.7%) | Reference | | | |  |  |  |
|  | 18-19 | 36 (19.1%) | 0.161 (0.314) | 0.608 | 1.174 | [0.635, 2.172] |  |  |  |
| Gender^+^ | Male | 32 (19.5%) | -0.277 (0.311) | 0.374 | 0.758 | [0.412, 1.395] |  |  |  |
|  | Female | 40 (20.5%) | Reference | | | |  |  |  |
| Ethnicity | Brahmin/Chhetri | 41 (16.1%) | -0.320 (0.313) | 0.307 | 0.726 | [0.394, 1.341] |  |  |  |
|  | Others | 29 (27.4%) | Reference | | | |  |  |  |
| Religion | Hindu | 68 (19.2%) | -0.999 (0.899) | 0.267 | 0.368 | [0.063, 2.146] |  |  |  |
|  | Others | 2 (28.6%) | Reference | | | |  |  |  |
| Grade | 11 | 39 (21.4%) | Reference | | | |  |  |  |
|  | 12 | 35 (19.6%) | -0.102 (0.310) | 0.741 | 0.903 | [0.492, 1.657] |  |  |  |
| Education System | Public | 37 (30.8%) | Reference | | | |  |  |  |
|  | Private | 37 (15.4%) | -1.124 (0.319) | <0.001 | 0.325 | [0.174, 0.608] |  |  |  |
| Current living condition | Living with family | 46 (16.8%) | -0.707 (0.327) | 0.030 | 0.493 | [0.260, 0.936] |  |  |  |
|  | Others | 24 (27.6%) | Reference | | | |  |  |  |
| Mother’s* education | No formal schooling | 21 (23.3%) | Reference | | | |  |  |  |
|  | Formal schooling | 48 (18.0%) | 0.104 (0.367) | 0.776 | 1.110 | [0.540, 2.281] |  |  |  |
| Mother’s* occupation | Employed | 67 (19.0%) | 0.113 (0.306) | 0.712 | 1.119 | [0.614, 2.040] |  |  |  |
|  | Unemployed | 2 (40.0%) | Reference | | | |  |  |  |
| Father’s** education | No formal schooling | 8 (21.1%) | Reference | | | |  |  |  |
|  | Formal schooling | 60 (19.2%) | 0.219 (0.501) | 0.662 | 1.245 | [0.466, 3.327] |  |  |  |
| Father’s** occupation | Employed | 65 (19.5%) | 0.362 (0.704) | 0.608 | 1.436 | [0.361, 5.709] |  |  |  |
|  | Unemployed | 3 (18.8%) | Reference | | | |  |  |  |
| Parents’*** chronic illness | Yes | 21 (30.0%) | 0.751 (0.341) | 0.028 | 2.119 | [1.086, 4.133] |  |  |  |
|  | No | 52 (18.1%) | Reference | | | |  |  |  |
| Parent’s*** smoking habit | Yes | 15 (28.3%) | 0.177 (0.436) | 0.685 | 1.193 | [0.507, 2.808] |  |  |  |
|  | No | 58 (19.0%) | Reference | | | |  |  |  |
| Parent’s*** smokeless tobacco | Yes | 22 (25.0%) | -0.228 (0.372) | 0.541 | 0.796 | [0.384, 1.652] |  |  |  |
|  | No | 51 (18.9%) | Reference | | | |  |  |  |
| Parent’s*** drinking habit | Yes | 30 (30.6%) | 1.004 (0.365) | 0.006 | 2.728 | [1.333, 5.584] |  |  |  |
|  | No | 43 (16.5%) | Reference | | | |  |  |  |

Table 8

| Characteristics | Categories | Current Smoker n (%) | B (SE) | p-value | OR  Exp(B) | 95% CI |  |  |  |
| --- | --- | --- | --- | --- | --- | --- | --- | --- | --- |
| Age | 16-17 | 17 (9.8%) | Reference | | | |  |  |  |
|  | 18-19 | 27 (14.4%) | 0.114 (0.426) | 0.789 | 1.121 | [0.487-2.581] |  |  |  |
| Gender^+^ | Male | 38 (23.2%) | 2.428 (0.507) | <0.001 | 11.336 | 4.196-30.623] |  |  |  |
|  | Female | 6 (3.1%) | Reference | | | |  |  |  |
| Ethnicity | Brahmin/Chhetri | 24 (9.4%) | -0.732 (0.425) | 0.085 | 0.481 | [0.209-1.105] |  |  |  |
|  | Others | 20 (18.9%) | Reference | | | |  |  |  |
| Religion | Hindu | 42 (11.9%) | -2.524 (1.123) | 0.025 | 0.080 | [0.009-0.724] |  |  |  |
|  | Others | 2 (28.6%) | Reference | | | |  |  |  |
| Grade | 11 | 20 (11.0%) | Reference | | | |  |  |  |
|  | 12 | 24 (13.4%) | 0.162 (0.400) | 0.685 | 1.176 | [0.537-2.579] |  |  |  |
| Education System | Public | 18 (15.0%) | Reference | | | |  |  |  |
|  | Private | 26 (10.8%) | -0.511 (0.449) | 0.255 | 0.600 | [0.249-1.447] |  |  |  |
| Current living condition | Living with family | 32 (11.7%) | -0.377 (0.451) | 0.402 | 0.686 | [0.284-1.658] |  |  |  |
|  | Others | 12 (13.8%) | Reference | | | |  |  |  |
| Mother’s* education | No formal schooling | 5 (5.6%) | Reference | | | |  |  |  |
|  | Formal schooling | 38 (14.2%) | 1.542 (0.612) | 0.012 | 4.675 | [1.408-15.523] |  |  |  |
| Mother’s* occupation | Employed | 42 (11.9%) | -0.321 (0.419) | 0.443 | 0.725 | [0.319-1.649] |  |  |  |
|  | Unemployed | 1 (20.0%) | Reference | | | |  |  |  |
| Father’s** education | No formal schooling | 5 (13.2%) | Reference | | | |  |  |  |
|  | Formal schooling | 38 (12.2%) | -0.692 (0.682) | 0.310 | 0.501 | [0.132-1.904] |  |  |  |
| Father’s** occupation | Employed | 42 (12.6%) | 2.327 (1.514) | 0.124 | 10.245 | [0.527-199.095] |  |  |  |
|  | Unemployed | 1 (6.3%) | Reference | | | |  |  |  |
| Parents’*** chronic illness | Yes | 6 (8.6%) | -0.400 (0.534) | 0.454 | 0.670 | [0.235-1.908] |  |  |  |
|  | No | 37 (12.8%) | Reference | | | |  |  |  |
| Parent’s*** smoking habit | Yes | 13 (24.5%) | 1.117 (0.551) | 0.043 | 3.055 | [1.037-8.997] |  |  |  |
|  | No | 30 (9.8%) | Reference | | | |  |  |  |
| Parent’s*** smokeless tobacco | Yes | 20 (22.7%) | 1.010 (0.438) | 0.021 | 2.745 | [1.163-6.479] |  |  |  |
|  | No | 23 (8.5%) | Reference | | | |  |  |  |
| Parent’s*** drinking habit | Yes | 16 (16.3%) | -0.415 (0.478) | 0.385 | 0.661 | [0.259-1.684] |  |  |  |
|  | No | 27 (10.4%) | Reference | | | |  |  |  |

Table 9

| Characteristics | Categories | Current Alcohol User n (%) | B (SE) | p-value | OR  Exp(B) | 95% CI |  |  |  |
| --- | --- | --- | --- | --- | --- | --- | --- | --- | --- |
| Age | 16-17 | 11 (6.4%) | Reference | | | |  |  |  |
|  | 18-19 | 26 (13.8%) | 0.557 (0.469) | 0.235 | 1.745 | [0.696-4.377] |  |  |  |
| Gender^+^ | Male | 34 (20.7%) | 2.647 (0.641) | <0.001 | 14.112 | [4.015-49.600] |  |  |  |
|  | Female | 3 (1.5%) | Reference | | | |  |  |  |
| Ethnicity | Brahmin/Chhetri | 21 (8.2%) | -0.781 (0.453) | 0.085 | 0.458 | [0.188-1.113] |  |  |  |
|  | Others | 16 (15.1%) | Reference | | | |  |  |  |
| Religion | Hindu | 36 (10.2%) | -1.430 (1.322) | 0.279 | 0.239 | [0.018-3.191] |  |  |  |
|  | Others | 1 (14.3%) | Reference | | | |  |  |  |
| Grade | 11 | 14 (7.7%) | Reference | | | |  |  |  |
|  | 12 | 23 (12.8%) | 0.077 (0.417) | 0.853 | 1.080 | [0.477-2.444] |  |  |  |
| Education System | Public | 14 (11.7%) | Reference | | | |  |  |  |
|  | Private | 23 (9.5%) | -0.240 (0.491) | 0.625 | 0.787 | [0.301-2.058] |  |  |  |
| Current living condition | Living with family | 31 (11.3%) | 0.195 (0.520) | 0.707 | 1.216 | [0.439-3.368] |  |  |  |
|  | Others | 6 (6.9%) | Reference | | | |  |  |  |
| Mother’s* education | No formal schooling | 6 (6.7%) | Reference | | | |  |  |  |
|  | Formal schooling | 31 (11.6%) | 0.551 (0.576) | 0.338 | 1.736 | [0.562-5.363] |  |  |  |
| Mother’s* occupation | Employed | 35 (9.9%) | -0.318 (0.432) | 0.461 | 0.728 | [0.312-1.696] |  |  |  |
|  | Unemployed | 2 (40.0%) | Reference | | | |  |  |  |
| Father’s** education | No formal schooling | 2 (5.3%) | Reference | | | |  |  |  |
|  | Formal schooling | 33 (10.6%) | 0.554 (0.850) | 0.515 | 1.741 | [0.329-9.218] |  |  |  |
| Father’s** occupation | Employed | 34 (10.2%) | 1.476 (1.271) | 0.246 | 4.374 | [0.362-52.806] |  |  |  |
|  | Unemployed | 1 (6.3%) | Reference | | | |  |  |  |
| Parents’*** chronic illness | Yes | 8 (11.4%) | -0.001 (0.509) | 0.998 | 0.999 | [0.368-2.709] |  |  |  |
|  | No | 29 (10.1%) | Reference | | | |  |  |  |
| Parent’s*** smoking habit | Yes | 9 (17.0%) | 0.138 (0.596) | 0.816 | 1.148 | [0.357-3.691] |  |  |  |
|  | No | 28 (9.2%) | Reference | | | |  |  |  |
| Parent’s*** smokeless tobacco | Yes | 15 (17.0%) | 0.528 (0.465) | 0.256 | 1.695 | [0.682-4.212] |  |  |  |
|  | No | 22 (8.1%) | Reference | | | |  |  |  |
| Parent’s*** drinking habit | Yes | 16 (16.3%) | 0.237 (0.461) | 0.607 | 1.268 | [0.513-3.133] |  |  |  |
|  | No | 21 (8.1%) | Reference | | | |  |  |  |

Table 10

| Characteristics | Categories | Current Smokeless Tobacco Use n (%) | B (SE) | p-value | OR  Exp(B) | 95% CI |  |  |  |
| --- | --- | --- | --- | --- | --- | --- | --- | --- | --- |
| Age | 16-17 | 12 (6.9%) | Reference | | | |  |  |  |
|  | 18-19 | 19 (10.1%) | -0.154 (0.466) | 0.742 | 0.858 | [0.344, 2.138] |  |  |  |
| Gender^+^ | Male | 28 (17.1%) | 2.557 (0.646) | <0.001 | 12.896 | [3.636, 45.740] |  |  |  |
|  | Female | 3 (1.5%) | Reference | | | |  |  |  |
| Ethnicity | Brahmin/Chhetri | 22 (8.6%) | 0.037 (0.492) | 0.940 | 1.038 | [0.395, 2.723] |  |  |  |
|  | Others | 9 (8.5%) | Reference | | | |  |  |  |
| Religion | Hindu | 30 (8.5%) | -1.640 (1.284) | 0.201 | 0.194 | [0.016, 2.403] |  |  |  |
|  | Others | 1 (14.3%) | Reference | | | |  |  |  |
| Grade | 11 | 14 (7.7%) | Reference | | | |  |  |  |
|  | 12 | 17 (9.5%) | 0.093 (0.435) | 0.832 | 1.097 | [0.468, 2.572] |  |  |  |
| Education System | Public | 10 (8.3%) | Reference | | | |  |  |  |
|  | Private | 21 (8.7%) | -0.322 (0.495) | 0.515 | 0.725 | [0.275, 1.912] |  |  |  |
| Current living condition | Living with family | 24 (8.8%) | 0.086 (0.519) | 0.869 | 1.089 | [0.394, 3.011] |  |  |  |
|  | Others | 7 (8.0%) | Reference | | | |  |  |  |
| Mother’s* education | No formal schooling | 5 (5.6%) | Reference | | | |  |  |  |
|  | Formal schooling | 25 (9.4%) | 0.238 (0.579) | 0.681 | 1.269 | [0.408, 3.948] |  |  |  |
| Mother’s* occupation | Employed | 29 (8.2%) | -0.290 (0.441) | 0.511 | 0.748 | [0.315, 1.776] |  |  |  |
|  | Unemployed | 1 (20.0%) | Reference | | | |  |  |  |
| Father’s** education | No formal schooling | 2 (5.3%) | Reference | | | |  |  |  |
|  | Formal schooling | 28 (9.0%) | 0.282 (0.827) | 0.733 | 1.326 | [0.262, 6.713] |  |  |  |
| Father’s** occupation | Employed | 29 (8.7%) | 0.718 (1.165) | 0.538 | 2.050 | [0.209, 20.126] |  |  |  |
|  | Unemployed | 1 (6.3%) | Reference | | | |  |  |  |
| Parents’*** chronic illness | Yes | 6 (8.6%) | 0.072 (0.522) | 0.891 | 1.075 | [0.386, 2.992] |  |  |  |
|  | No | 24 (8.3%) | Reference | | | |  |  |  |
| Parent’s*** smoking habit | Yes | 5 (9.4%) | -0.132 (0.661) | 0.842 | 0.877 | [0.240, 3.202] |  |  |  |
|  | No | 25 (8.2%) | Reference | | | |  |  |  |
| Parent’s*** smokeless tobacco | Yes | 9 (10.2%) | 0.103 (0.509) | 0.840 | 1.108 | [0.409, 3.005] |  |  |  |
|  | No | 21 (7.8%) | Reference | | | |  |  |  |
| Parent’s*** drinking habit | Yes | 12 (12.2%) | 0.415 (0.472) | 0.379 | 1.515 | [0.600, 3.825] |  |  |  |
|  | No | 18 (6.9%) | Reference | | | |  |  |  |
