# Supplementary material for: Behavioural risk factors for cardiovascular diseases among adolescents of secondary school in Tulsipur Sub-Metropolitan City, Nepal: A cross-sectional study
Source: PLoS One. 2025 Sep 11;20(9):e0313943. doi: 10.1371/journal.pone.0313943 (PMC12425188; doi:10.1371/journal.pone.0313943)
Supplement: Attachment — Submitted filename: Rebuttal letter_BB edits_17th Feb_2025.docx [file pone.0313943.s005.docx]

**Rebuttal letter to reviewers comments Manucript ID PONE-D-24-49797**

17^th^ February 2025

Dear Dr. Dhital,

Academic editor, PLOS One

We thank you and the reviewers for the constructive comments on our manuscript, “Behavioural risk factors for cardiovascular disease among adolescents of secondary level school in a sub-metropolitan setting in Nepal: A cross-sectional study (PONE-D-24-49797).” We have carefully addressed all comments and revised the manuscript accordingly. Below is a point-by-point response to each comment.

**Recommendations for Revision from Reviewer 1** :
1. Enhance Methodological Rigor: Addressing self-reporting limitations with more objective measures, if feasible, or by discussing potential biases in-depth, could enhance the study's reliability.

**Response:**  Thank you for your insightful comments. We have revised the limitation section accordingly and added the potential bais in the revised manuscript. Changes can be seen in page no 20, line no 243-249.

2. Statistical Refinement: Adding multivariate analyses would help substantiate causal inferences and control for confounding variables, lending greater credibility to findings.

**Response:** We have incorporated the reviewer’s suggestion and conducted the multivariate analysis (Binary logistic regression) along with the Chi square test to ensure the robustness of association. The multivariate analysis data analysis is presented in table 5and findings are updated throughout the manuscript in the result ( page 16-17 and Line no 170-178) and discussion as well.

3. Data Visualization: Incorporate more visual data representations, particularly for associations impacted by socio-demographic factors, to improve readability and impact

**Response:** Thanks you for the suggestion. We have substituted table 1 with the bar chart displaying the prevalence of behavioral risk factors in adolescents and binary logistic regression table 5) was added to results section displaying significance of association and Odds ratio.

**Reviewer #2: Comments**

1. In the title sub-metropolitan setting is misleading as this study is conducted in Tulsipur sub-metropolitan city only. Abstract doesn't says what is the study population is?

**Response**: Thank you for your valuable remarks. We have revised the title to specifically mention "Tulsipur Sub-Metropolitan City" instead of the general term "sub-metropolitan setting" for clarity. We have also clarified the study population in the abstract ( line 17-18)

2. Does your study really used simple random lottery method to select students from the school. If so how have you reach out the specific students to collect information? If your study is stratified proportionate sampling, you should present the results stratified by rural and urban schools and further stratified by 11 and 12th grade. You need to be cautious while merging two groups and estimate the proportion for all population.
   Please add details on how you reach out to the students.

**Response**: Thank you for providing opportuntiy to clarify our sampling approach. A stratified proportionate sampling method was employed using sampling frame obtained from municipal education section. The two strata were defined based on schools’ affiliation (Public/Private), with the sample size proportionally distributed based on the student population in each stratum. Two public and two private secondary schools were randomly selected. The students were divided into grades 11 and 12, with a proportional distribution according to age. Within each school, students were selected through a simple random lottery method using a random number generator. The student’s records and details of each selected school were obtained from the school administration. Each student were assigned a unique identification number. Based on proportionate sample of selected schools, Microsoft excel was used to generate a predefined and non-repeating number. The randomly generated numbers were cross-verified with the student details. Then after, selected students were invited to participate in study. If selected students didn’t provide consent or were absent, alternative students were taken through additional random numbers generated. These information are clarified in the revised manuscript (page no 4-5, line no 86-98)

There was stratification by public and private schools, then proportionate sampling was taken from 11^th^ and 12^th^ grade but not stratified by rural and urban schools and which is included as a limitation of the study in the limitation section.

3. Line 79: Missing inline citation

**Response:** We have inserted citation.

4. Line number 94-96: Who or how many people collected data and who supervised whom? It needs to be clarified.

**Respone:** The data was collected by the first and second author (SB and BP) who were supervised by the senior author (BB and PT). We have calrified our approach in the revised manuscript.

5. Line 103: what are you calculating confidence interval of ??

**Response**: The 95% confidence interval was calculated to measure the precision of the estimated associations in the chi-square test.

6. For those with age less than 18 what have you done.

**Response**: Both written parental consent and student’s consent were taken for age less than 18 and those students with aged 18 or above provided their written consent. On the first day, Parents were provided with parental consent form through students, then only if agreed by parents for involvement in study, students were provided with written consent form. This has been clarified in the revised manuscript ( page 6, line 117-121)

7. Statistical analysis part misses about SD that you calculated in results

**Response: Thank you.** This issue has been addressed at data processing and analysis section. ( page no 7, line no 127)

8. Line 117: Please keep all details of participants characteristics within manuscript.

**Response:** We have moved the table of characteristics of participants from the supplementary file to the manuscript (Table 1page 7, line 136).

9. Calculate % and its 95% CI rather than just point estimation (use Wilson method or other appropriate method to calculate 95% CI)
10. **Response:** Adjusted Odds ratio has been calculated by using binary logistic regression analysis.

     Results from supplementary tables are in the discussion. Please keep whatever you discuss in the main text

**Response:** Data discussed in the manuscript are presented within the manuscript; only further information is provided at the supplementary file section.

11. Include number of missing data each variables if missing.

**Response:** Thank you for your suggestion to include number of missing data each variables if missing. We didn’t find any missing values of variables after carefully reviewing the dataset, except for the parent’s characteristics that has been clarified in the footnote of Table 1.

12. Reference should be formatted as per journal requirement

**Response:** Thank you, we have formatted as per journal requirement

13. requires extensive grammar correction

**Response:** Thank you. We have revised and proof read the manuscript for the language and grammatical errors.

We sincerely appreciate the opportunity to revise and resubmit our manuscript. We are confident that the quality of the revised manuscript has significantly improved and now meets the expectations of the reviewers and the journal.

Thank you for your time and consideration.

Sincerely,

Sita Bista
