# Supplementary material for: Behavioural risk factors for cardiovascular diseases among adolescents of secondary school in Tulsipur Sub-Metropolitan City, Nepal: A cross-sectional study
Source: PLoS One. 2025 Sep 11;20(9):e0313943. doi: 10.1371/journal.pone.0313943 (PMC12425188; doi:10.1371/journal.pone.0313943)
Supplement: Attachment — Submitted filename: Rebuttal letter _12th July.docx [file pone.0313943.s006.docx]

**Rebuttal letter to reviewers comments Manuscript ID PONE-D-24-49797**

12^th^ July, 2025

Dear Dr. Adhikari,

Academic editor, PLOS One

We thank you and the reviewers for the constructive comments on our manuscript, “Behavioural risk factors for cardiovascular disease among adolescents of secondary level school in a sub-metropolitan setting in Nepal: A cross-sectional study (PONE-D-24-49797).” We have carefully addressed all comments and revised the manuscript accordingly. Below is a point-by-point response to each comment.

**Recommendations for Revision**

**Academic Editor**

1.In line 82-85, N=5262, is this total of school going adolescent of Tulsipur, or of only 16-19? **Response:** Thank you for your comment. This is the total population of secondary level school adolescents of age between 16-19 studying in only grade 11 and 12, which is clarified in the manuscript( page 5, line 81)

2. Line 104-105, What was the pretested sample population, and place, pls specify.

**Response:** Thank. We have provided details of the pretesting in the revised manuscript as follows

Pretesting was conducted among 37 students from Ghorahi sub-metropolitan city, a location with similar characteristics to the study area.(page 6 , line 103-105). The pre-testing data was not included in final analysis.

3. Table 2, 3, 4; percent total was observed, but columwise p-value does not comply with, so columwise % total may be better interpretable?!

**Response:** Thank you for your suggestion. We have updated all tables with the column wise percentage and total as evidenced in revised table Table 2, 3 and 4.

4. Table 5 shows the UOR (?), and the list of regressors are too many and poorly interpretable, such as mother's education and ethnicity may be just the confounders for processed food consumption. So better to reduce the risk factors with further adjustment with modelling and so, better interpretable to discuss more contextually. And then, re-discuss as of the new results.

**Response:** Thank you for your suggestion. We have conducted further analysis adjusting for potential confounders using a multivariable logistic model and now present both **Adjusted Odds Ratios (AORs)** and Unadjusted Odds Ratios (UORs) in the revised Table 5 (page 21, line no 181). The discussion section has also been updated to reflect and contextualize these updated findings accordingly.

**Reviewer #3:**

**Title and Abstract**
Comment:
1. The phrase “affecting one-third of adolescents” in the abstract may be misleading. Please clarify whether this refers to actual/clinical CVD or its risk factors. (Same comment for introduction part; Line no 43)

**Response:** Thank you for your valuable remarks. We acknowledge that the original phrasing may have caused confusion. The statement was intended to refer to **cardiovascular disease (CVD)** affecting one-third of adult population. We have revised the sentence in both the abstract (Line no.13, page no.2) and the introduction (Line no 40, page no. 3) to clarify this confusion.

2. “Schoolswere” has a typo

**Response:** Thank you. We have edited it. (Line no 18, page no.2)

3. Spacing and formatting inconsistent on Key words section (It applies to the whole manuscript).

**Response:** We appreciate for highlighting the formatting issue. We have carefully reviewed and formatted the whole manuscript including spacing of the Keywords section.

**Introduction**
Comment:
1. The paragraph shifts abruptly from global data to adolescent.
Suggestion: It would be easier to understand if the data flowed from global to regional to national and local.

**Response:** Thank you for the insightful suggestion. We have revised the paragraph to enhance clarity and improve the flow, presenting the data in a logical sequence from global to regional to national levels, while maintaining a focus on the global burden and its relevance to adolescents. Unfortunately, sufficient data on the local burden are not available. (see changes page 3, line 38-46)

**Method**
1. What was the mechanism of identifying students with a history of CVDs or other chronic diseases (any students found during data collection if yes how)? Please mentioned it.

**Response:** Thank you for your valuable comment. All data were based on the students’ self-reported responses regarding their history of cardiovascular disease (CVD). None of the participants reported having a history of CVDs or other chronic conditions at the time of data collection.

2. The manuscript mentions the assessment of cardiovascular disease (CVD) risk behaviors among adolescents. The authors need to clarify whether any procedures were in place to ethically manage participants who were identified as being at heightened risk during the study.

**Response:** Thank you for this important observation. While our study did not involve clinical assessment or diagnosis of individual cardiovascular risk, we acknowledge the ethical responsibility when identifying high-risk behaviours among adolescents. To address this, we shared the de-identified and aggregated findings with the relevant municipal and school authorities to raise awareness about the high prevalence of major CVD risk factors and the importance of school-based health education program. As noted in the conclusion, this highlights an area for future research and opportunity for school based intervention. However, due to limited resources and observational nature of the study, we were not able to offer individual counseling or follow-up for participants identified as having potentially high CVD risk behaviors.

**Result**
1. author used asterisks (*) at multiples places, used different indicators (eg #) with clear footnotes and explanations (it applies to the other sections of results eg table no 3, 4)

**Response:** We appreciate your suggestions. We have revised the use of footnote indicators across all applicant tables, including Tables 1, 2, 3 and 4 to enhance clarity and maintain consistency throughout the manuscript.

2. Spelling error (line no 146)

**Response:** Thank you. We have corrected the errors on line 148, page no.12 and proofread the revised manuscript

3. In the “Association between CVD Risk Factors and Socio-demographic Characteristics” section, the author needs to indicate the significant result within the table by using an indicator (e.g., 0.028*) and highlighting the result; it could help the reader.

**Response:** Thank you for your feedback. We have highlighted the significant result consistently across all tables using an indicator.

**Discussion**
1. In the first paragraph of the discussion, the author had summarized the findings, which can be more concise, and if the author put “what is going to be discussed,” it would be easier for readers. Then only start the discussion in another paragraph.

**Response:** Thank you. We have revised our discussion accordingly in the revised manuscript including breaking a paragraph in line no 190-194, page no. 25.

2. The author discussed it well, but it would be better to discuss separately to risks. For eg discuss physical activity in one single, calorie intake in another.

**Response:** Thank you. We have discussed the individual risk factors in individual paragraphs.

**Conclusion/Recommendation**
1. The word used by author “targeted intervention” sounds vague, what types of interventions and to whom/who are the high risk group? Could it be mentioned, and it will be more specific.

**Response:** We have clarified the possible targeted intervention in the revised manuscript as follows:

Targeted interventions may include age-appropriate school health education on cardiovascular risk factors integrated into the curriculum; promotion of daily physical activity; restrictions on processed fast foods and sugary beverages in school cafeterias; regular health screenings (e.g., blood pressure, BMI, blood glucose); mental health workshops and counseling sessions; and referral of high-risk children to health professionals. (line 272-277, page 29)

We sincerely appreciate the opportunity to revise and resubmit our manuscript. We are confident that the quality of the revised manuscript has significantly improved and now meets the expectations of the reviewers and the journal.

Thank you for your time and consideration.

Sincerely,

Sita Bista
